# The Law’s Charlatanry

**Published:** 1880-10

**Authors:** Ausburn Towner


### THE LAW'S CHARLATANRY.

AUSBURN TOWNER.

### I.

I have, heretofore, in the BISTOURY pointed out what might be called the charlatanry of the law in regard to women, and the examination of that subject has led me to believe that the law itself has very much of humbug about it. By the term Law, I mean, not Statute Law, for that has determined boundaries and a fixed quantity, but what is called common Law, rules of conduct that have precedent for their foundation, or a decision or decisions made by a Justice, it may be last week or last century, or a custom so old that "the memory of man runneth not to the contrary." These are sometimes dignified with the name of "principles," "maxims" or "axioms."

As mere abstractions, there may be such things, two and two will always make four, and a body will fall to the ground at a rate of speed that can be accurately and definitely determined before hand, but there never was a practical question so precisely like another practical question; there never was a series of facts so precisely paralleled by another series of facts that what was said or determined of the first can be said or determined of the second. A demonstration of this we see every day. What else can be inferred than that no two questions are alike, when we constantly hear arguments before a Justice, in which one lawyer refers to 44th Wendell, page 672 where Justice A—observed so and so, and the other lawyer says that in the 22nd, of Wheaton, page 283, Justice B—decides in an opposite way and then the court agrees that it may be as the lawyers say, but in the 33rd of Comstock, Justice C— held entirely unlike either of the other two ? The decisions, it will often be found on examination, are different because they concern questions that though seeming to be alike are really unlike and unlike the one that is being argued. I often feel like inquiring, when such references are made in the course of an argument: What of it? It is not what Justice A— or Justice B— or Justice C— held years and years ago, but what do you yourself say, Mr. Justice as to the question or the case before you? They decided their questions according to the light they had from them. Your position should require of you to do the same or retire from it. And because each question or case is distinct by itself, unlike any other in its import and surroundings, so let each question or case be decided by itself, without regard to what has gone before or what is to come after.

To a disinterested observer, the height of the absurd and idiotic is nowhere on earth seen to such entire perfection as in a Court of Law. Much of this comes from a slavish adherence to precedent. I doubt if there is ever a case tried these days, in which there is not that which would make a thoughtful man believe that while everything else in the way of human endeavor is becoming simplified and so, advancing toward perfection the Law is retrograding and getting more and more confused and muddy. I have a number of illustrations to present of the point at which I aim; illustrations that I have not sought for, nor isolated or infrequent ones, but arising hap hazard to my observation on careless glances at the reports of trials, the difficulty being rather in the making of judicious selections than in the want of them.

In the indictment on the second trial of the Rev. Herbert H. Hayden in Connecticut for the murder of Mary Stannard, the girl was once named Mary Hayden, an error clearly of the copyist. The Justice governed, of course, by what some one else had said perhaps three centuries ago, decided that the mistake was a fatal one; that the indictment could not be amended; must be quashed and a new one drawn. The decision and the change cost the county upwards of $2,000 and a delay of a week. A disinterested observer would be excusable for turning himself into an animated interrogation point in regard to such a decision. It was as clear as the rarest day in June what was meant, and who was meant. A correction, the simplest and most straight forward way out of the difficulty, if there was a real difficulty, could not have prejudiced the accused person, nor benefited the prosecution. It seems to me that the natural, just and rational way would have been to have run a pen through the name of Hayden; to have written Stannard above it and then to have drawn the first name in the panel. Setting aside the absurd and mischievous notion of precedent, either as the question was affected by the past or as it affected the future and the person most "learned in the Law" would find it extremely difficult to give one substantial reason why the course I suggest would not have been, in every respect, for the best.

In that same neighborhood in Connecticut, and at about the same time, there was a German by the name of Bucholz, tried and convicted for the murder of his employer, another German named Schulte. There was no doubt of the guilt of the prisoner, from his own confession to a detective, yet he came near going scot free because of the difficulty in the construction of a recent statute of the State, providing, "that there shall not be a conviction for murder in the first degree except on the testimony of two eye witnesses or its equivalent." Stop a minute there reader and see if you can conjure up where any difference of opinion could arise. Applying the method of reasoning that obtains in every other discussion except one concerning the Law, and it is ten to one that you would have to give it up as you would a blind conundrum. The argument arose as to what fact the statute required that the eye witnesses should testify! It is not easy to imagine otherwise intelligent men arguing upon such a point. The platitudes of a winter's debating society in some school-house in the woods, rise to dignity beside such peurile discussions.

On Aug. 2, 1879, Mary H. Reeve, wife of a New York lawyer, was on her way to Cable's on Coney Island, to the Ocean Hotel. In order to get to her destination she had to walk along a plank road belonging to the Prospect Park and Coney Island railroad company, and while going over this walk, one of the planks on which she stepped, raised up. She fell through the opening, a distance of several feet and in falling, broke her nose and sustained severe injuries upon her body and legs. She sued the company to recover damages and her husband also sued for loss of services. The railroad company's lawyer made a motion before a Judge in Chambers for an order compelling Mrs. Reeve to submit to a physical examination at the hands of some competent physician to be named by the Court. He supported his motion by citing a case in Howard's Reports and an Iowa case, as showing that the Judge had power. The motion was opposed by Mrs. Reeve's lawyer who contended that the court had no power and that the exercise of it would be trespass, citing in support of his argument a number of cases both in this country and in England. He contended also, that if the court had the power, it would not exercise it in this instance, because a physical examination would not assist science, inasmuch as all evidence of wounds, cuts, scars and injuries had passed away and the parts were restored to their normal condition. The Judge said that the question was a very important (!) one and reserved his decision.

This is a sharp illustration of the point I would make. No reasonable man will question the justice or propriety of the request made by the lawyer for the railroad company. If the truth was to be got at, and if a suit at law is for that purpose, how better could it be reached than for some competent person to make a physical examination and determine the extent of the hurts and injuries complained of? What need of referring to what happened in Iowa to sustain a proposition so self-evident and so capable of standing by itself? But the lady's lawyer said: No. It is some time since the accident happened, and although it caused much pain and more or less expense, we can show that no scars are left and a physical examination would therefore be useless. He is thoroughly reasonable also, and what need to refer to what some Justice has held in England years ago? And now the Judge utters his dictum. The question is an important one, beyond his immediate comprehension and he reserves his decision! Is not this silly? It certainly would be, applied to any other of the affairs of mankind except those bound in the law. Again, I repeat, it would be no easy matter, for one howsoever learned he may be in the law, to demonstrate the importance of the question, as a question, as claimed by the court, when it is stripped of its element of precedent.

When Joseph A. Blair was tried in New Jersey, last fall for shooting John Armstrong, his coachman, the defence introduced a witness to testify to certain statements made by an important witness for the prosecution. Ophelia Dyer a servant in the family of the prisoner at the time of the shooting, was the witness for the prosecution. The defense called George M. Dusenberry in whose service Ophelia entered after quitting Blair's house. The lawyer for the defence asked Dusenberry if Ophelia had ever told him that before the shooting, Armstrong had said that if any of the Blairs came to the barn he would blow their brains out. The question had hardly been propounded before the district attorney was on his feet with an objection, the substance of which was that Ophelia Dyer had not sworn that she did not tell Dusenberry what the question intimated she had told him and so, the witness Dusenberry could not swear that she had told him so!* Or, in the pompous circumlocution of the Law, the witness Dusenberry, it was shown, had been called to contradict Ophelia Dyer further. The State claimed that she had made no statement as to what she had told the Dusenberrys and that no foundation for the contradiction had been laid! The defence thought she had. The Court was doubtful and it was concluded to put Ophelia on the stand again that she might make the statement it was desired to contradict! Ophelia swore that she had never said to the Dusenberrys the words already set forth and then Dusenberry contradicted her and swore that she had! This would be funnier than one of Gilbert's most "screaming farces," except in showing the painful degradation into which the Law has fallen.

This objection set forth above is a very common one, is made possible by decisions rendered years ago, when, perhaps, there were reasons for it, and can be heard almost any day in our court-rooms. The first thought it suggests to a disinterested listener or to a reflecting juror is, that the objector wants to suppress something; that he is not anxious for the whole truth to come out; and the second thought is, that it is all a species of boy's play, hardly consistent with the dignity that is supposed to sit enthroned in a court-room. Wise mandarins in China sit by the hour solemnly clinging to the strings of their kites. It is a precedent that has been handed down to them through a long series of years, and, custom upholding the action, no one laughs. Custom, it may be, would save the silliest act from its natural sequence, but it cannot save such questions and objections as I have noted, and they are only samples of a multitude, from the contempt that belongs to them, when they are properly set before the

* This would be laughable and sounds like a travesty or burlesque, except that in any other of the concerns of life but the law, it would certainly be the wickedest folly. One should possess the vocabulary of Thersites to characterize it fittingly and the multitude of similar displays that one constantly sees in a Court of Law.

apprehension of those not interested in their discussion.

There are other branches of this same topic, that can easily be illustrated, but of them and concerning a remedy for the gigantic abuse which The Law has become, I reserve for the next number of the BISTOURY.

